# Race and trial region changes in cancer clinical trials between 2013 and 2024

**DOI:** 10.3389/fmed.2026.1894533

**Published:** 2026-09-09

**Authors:** Satoshi Hirano, Keisuke Hanada, Hideki Maeda

**Affiliations:** 1 Regulatory Science, Graduate School of Pharmaceutical Science, Meiji Pharmaceutical University, Tokyo, Japan; 2 Department of Biostatistics, Faculty of Medicine, Wakayama Medical University, Wakayama, Japan

**Keywords:** clinical trial, enrollment, oncology, race, trial region

## Abstract

**Background:**

The latest changes in racial and geographical enrollment in cancer clinical trials in the 2020s have not been fully characterized, despite ongoing measures by the US Food and Drug Administration (FDA) to enhance diversity. We aimed to quantitatively elucidate the trial population of FDA-approved cancer drugs by race and trial region in 2013–2024.

**Methods:**

FDA drug approvals granted from January 2013 through December 2024 were reviewed by the FDA Oncology (Cancer)/Hematologic Malignancies Approval Notifications and FDA Application Review Files. Of 515 approval notifications screened, 92 eligible ones were analyzed. The primary endpoint was the ratio of each race and trial region among the datasets submitted for FDA approval.

**Results:**

Our analysis included 23,539 participants. Although the majority race has remained White, the proportion of White decreased from 83.2% in 2013–2016 to 68.9% in 2021–2024. The proportion of Asians and Asian trial region increased from 9.3% and 7.4% in 2013–2016 to 21.4% (95% CI, 20.53–22.35) and 19.7% (95% CI, 18.81–20.57) in 2021–2024, respectively. The proportion of Black people remained under 4%.

**Conclusion:**

From 2013 to 2016 to 2021–2024, Black people underrepresentation has persisted, whereas the enrollment of Asians at race level and participants from Asia at region level have increased.

## 1 Introduction

Racial diversity in clinical trials is a demographic characteristic that facilitates the interpretation of the difference in efficacy and safety of a drug among different races. However, racial minorities are less likely to enroll in cancer clinical trials in the US. Specifically, a cross-sectional population analysis of cancer clinical trials sponsored by the National Cancer Institute reported that Black people and Asian/Pacific Islanders accounted for 7.9% and 2.2% of the total enrollees in 2002, respectively (1).

The US Food and Drug Administration (FDA) and scientific experts have published several guidance documents to provide recommendations addressing measures to enroll a representative population with respect to specific factors (2–5). The FDA published a step-by-step guide titled “Collection of Race and Ethnicity Data in Clinical Trials” in 2005 and 2016, detailing the approach to enrolling population-representative samples (2). The guide provides recommendations for sponsors to enroll participants who reflect the demographics of clinically relevant populations with respect to age, gender, race, and ethnicity. Furthermore, the FDA published the “Enhancing the Diversity of Clinical Trial Populations - Eligibility Criteria, Enrollment Practices, and Trial Designs” as ultimate guidance in November 2020 (3). The American Association for Cancer Research (AACR) and national academies have also provided recommendations on strategies to ensure diversity in clinical study participation (4, 5). The AACR has indicated that racial minorities, including Black people/African Americans, are underrepresented in clinical trials relative to their respective cancer burdens in the US (4). Clinical trials also provide the opportunity to receive the newest treatments; therefore, access to clinical trials should be equitable for all patients (4). Similarly, Consensus Study Reports published by the National Academies of Sciences, Engineering, and Medicine have highlighted seven concerns due to a lack of representation in clinical trials. These concerns were the following: 1) compromised generalizability of clinical research findings to the entire US population; (2) cost of hundreds of billions of dollars; 3) potential hindrance to innovation and discoveries; 4) exacerbation of low accrual that leads to trial failures; 5) limited access to effective medical interventions; 6) diminished trust in the clinical research enterprise and medical establishment; and 7) worsening health disparities in the populations currently excluded or underrepresented in clinical trials and research.

However, the progress in enrolling racial minorities remains unclear. A race analysis of clinical trials leading to FDA Oncology drug approvals between 2008 and 2018 indicated that Black people are underrepresented in these trials relative to their expected proportion based on cancer incidence and mortality in the US (6). This study compared the racial composition of global trial participants with US population-based estimates to evaluate the representativeness of trial populations supporting FDA oncology approvals. Because clinical trial datasets submitted to the FDA support approval of medical products intended for use in the United States, US population-based estimates provide a relevant regulatory and public health context for interpreting racial representation in FDA approval datasets, particularly for historically underrepresented groups in the United States. In this context, the FDA issued a draft guideline titled “Diversity Action Plans to Improve Enrollment of Participants from Underrepresented Populations in Clinical Studies Guidance for Industry,” requiring sponsors to submit Diversity Action Plans for certain clinical studies in the form and manner specified by the guidance (7). The Diversity Action Plans aim to increase the enrollment of participants who are members of historically underrepresented populations in clinical studies to help improve the strength and generalizability of the evidence for the target population.

Several studies have examined racial disposition in cancer clinical trials before 2020. However, despite the ongoing measures of the FDA and researchers to enhance diversity in clinical trials, the most recent population changes in racial enrollment in the 2020s have not been fully characterized. Moreover, changes in clinical trial regions may be associated with changes in racial enrollment, which also remains unclear. A World Health Organization database study has demonstrated an annual increase in the number of clinical trials recruiting in Asia in the 2008–2017 period (8). Therefore, the present study aimed to quantitatively elucidate the trial population in FDA-approved drugs by race and trial region in clinical trials conducted from 2013 to 2024.

## 2 Materials and methods

Data analysis was performed according to the Preferred Reporting Items for Systematic Reviews and Meta-Analyses (PRISMA) statement guidelines (9). Data were manually collected by one person and independently reviewed by another individual. This study did not require institutional review board approval or informed patient consent, as it was based on publicly available information and did not involve patient records.

### 2.1 Data collection

**Figure 1:**
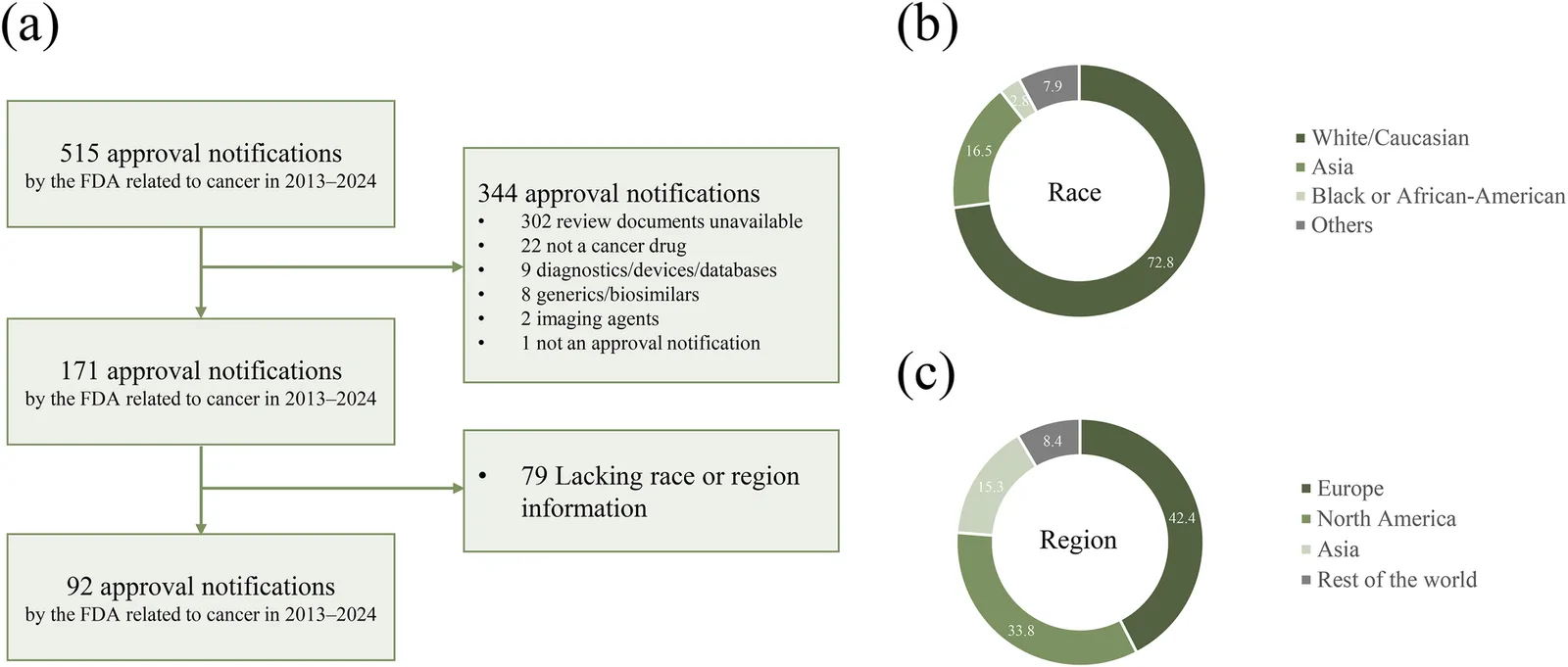
**(a)** Flow diagram of the search strategy. **(b)** Race distribution in 2013–2024. **(c)** Region distribution in 2013–2024.

The data collection scheme is presented in Figure 1. We reviewed FDA drug approvals granted from January 2013 through December 2024 from the FDA archives (10). The notifications about cancer drugs were included, whereas those that did not contain race and trial region information were excluded. Patient numbers by race and trial regions were collected from each FDA review document. When multiple trials supported FDA approval, the trial described as pivotal in the FDA review document was included in our analysis. If the demographics were not available in the pivotal study but were available in the integrated analysis including the pivotal study, the one in the integrated analysis was included in our analysis. The selected pivotal study number is provided in Supplementary Table S1.

### 2.2 Statistical analysis

The proportions of participants in each racial and geographic category were estimated using data extracted from FDA review documents for which the relevant race or trial-region information was available (Figure 1). For each racial or geographic category, trial-level aggregated counts were analyzed using grouped binomial logistic regression. Specifically, the number of participants in the category was treated as the event count, and the total number of participants in each trial was treated as the binomial denominator. A single model was fitted across all time periods for each racial or geographic category, with time period (2013–2016, 2017–2020, and 2021–2024) included as a categorical explanatory variable. This model-based approach was used to estimate proportions and corresponding 95% confidence intervals within a common analytical framework and to facilitate consistent subgroup comparisons while accounting for differences in trial sample sizes through the binomial denominators. For analyses according to FDA approval type, approval type and its interaction with time period were added to the model. Cancer type was analyzed similarly by replacing approval type with cancer type and including the corresponding interaction with time period. Estimated proportions and 95% confidence intervals were obtained by transforming the model estimates from the logit scale to the probability scale. Analyses were performed using the glm function in the stats package in R version 4.4.3 (11).

## 3 Results

There were 515 FDA approval notifications published between January 2013 and December 2024 in the FDA archives. In total, 171 notifications met the inclusion criterion. Of these, 79 were excluded as they met the exclusion criterion, leaving 92 approval notifications that were subsequently analyzed (Figure 1a).

The characteristics of the 92 approval notifications and integrated patient demographics in the data package for their approvals are presented in Table 1 and Supplementary Table S1. The 92 notifications were distributed as follows: 24% in 2013–2016, 40% in 2017–2020, and 36% in 2021–2024. Among these, notifications about accelerated approval accounted for 39%, while those regarding regular approval accounted for 61%. At the participant level, 23,539 participants were included in this analysis. Similar to the distribution at the notification level, the 23,539 participants were distributed as follows: 27% in 2013–2016, 40% in 2017–2020, and 33% in 2021–2024.

**Table 1:** Approval notifications and demographics of patients included in this analysis.

Characteristics	Approval notifications, No. (%)
All	92 (100)
Original approval	92 (100)
2013–2016	22 (24)
2017–2020	37 (40)
2021–2024	33 (36)
Accelerated approval	36 (39)
2013–2016	7 (8)
2017–2020	14 (16)
2021–2024	15 (16)
Regular approval	56 (61)
2013–2016	15 (16)
2017–2020	23 (25)
2021–2024	18 (20)

Characteristics	Trial participants, No. (%)
All	23,539 (100)
Original approval	23,539 (100)
2013–2016	6,358 (27)
2017–2020	9,359 (40)
2021–2024	7,822 (33)
Accelerated approval	5,366 (23)
2013–2016	1,232 (5)
2017–2020	1,818 (8)
2021–2024	2,316 (10)
Regular approval	18,173 (77)
2013–2016	5,126 (22)
2017–2020	7,541 (32)
2021–2024	5,506 (30)

The race and trial region disposition of all participants are reported in Figures 1b,c. The majority race was White, comprising 72.8% of the population. Asians accounted for 16.5% of all participants, while Black people accounted for 2.8%. At the trial region level, the total numbers in North America and the EU accounted for 76.2% of all trials (Europe, 42.4%; North America, 33.8%), while trials in Asia accounted for 15.3%.

**Figure 2:**
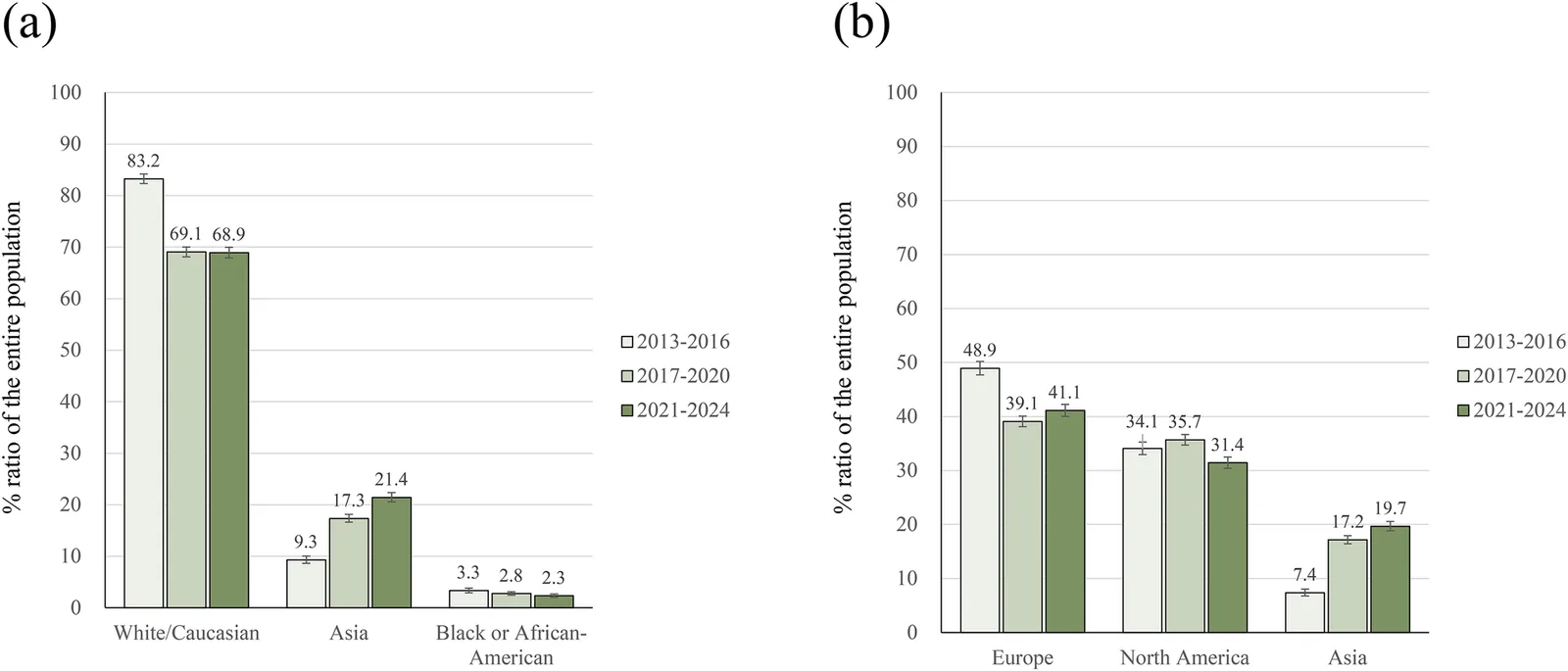
Race **(a)** and trial region **(b)** changes between 2013 and 2024 in cancer trials of FDA-approved drugs. Error bars represent 95% confidence intervals.

The population changes by race and trial region from 2013 to 2024 are presented in Figure 2. The majority race during this period has remained White; however, the proportion of White decreased from 83.2% (95% CI, 82.31–84.15) in 2013–2016 to 68.9% (95% CI, 67.90–69.95) in 2021–2024. In contrast, the proportion of Asians increased from 9.3% (95% CI, 8.591–10.02) in 2013–2016 to 21.4% (95% CI, 20.53–22.35) in 2021–2024. Black people accounted for 2.3%–3.3% in 2013–2024. At the trial region level, the number of participants in Europe decreased from 48.9% (95% CI, 47.70–50.16) in 2013–2016 to 41.1% (95% CI, 40.04–42.22) in 2021–2024, whereas those in North America remained between 31.4% (95% CI, 30.42–32.47) and 35.7% (95% CI, 34.73–36.67) in 2013–2024. Participants in Asia increased from 7.4% (95% CI, 6.74–8.03) in 2013–2016 to 19.7% (95% CI, 18.81–20.57) in 2021–2024.

**Figure 3:**
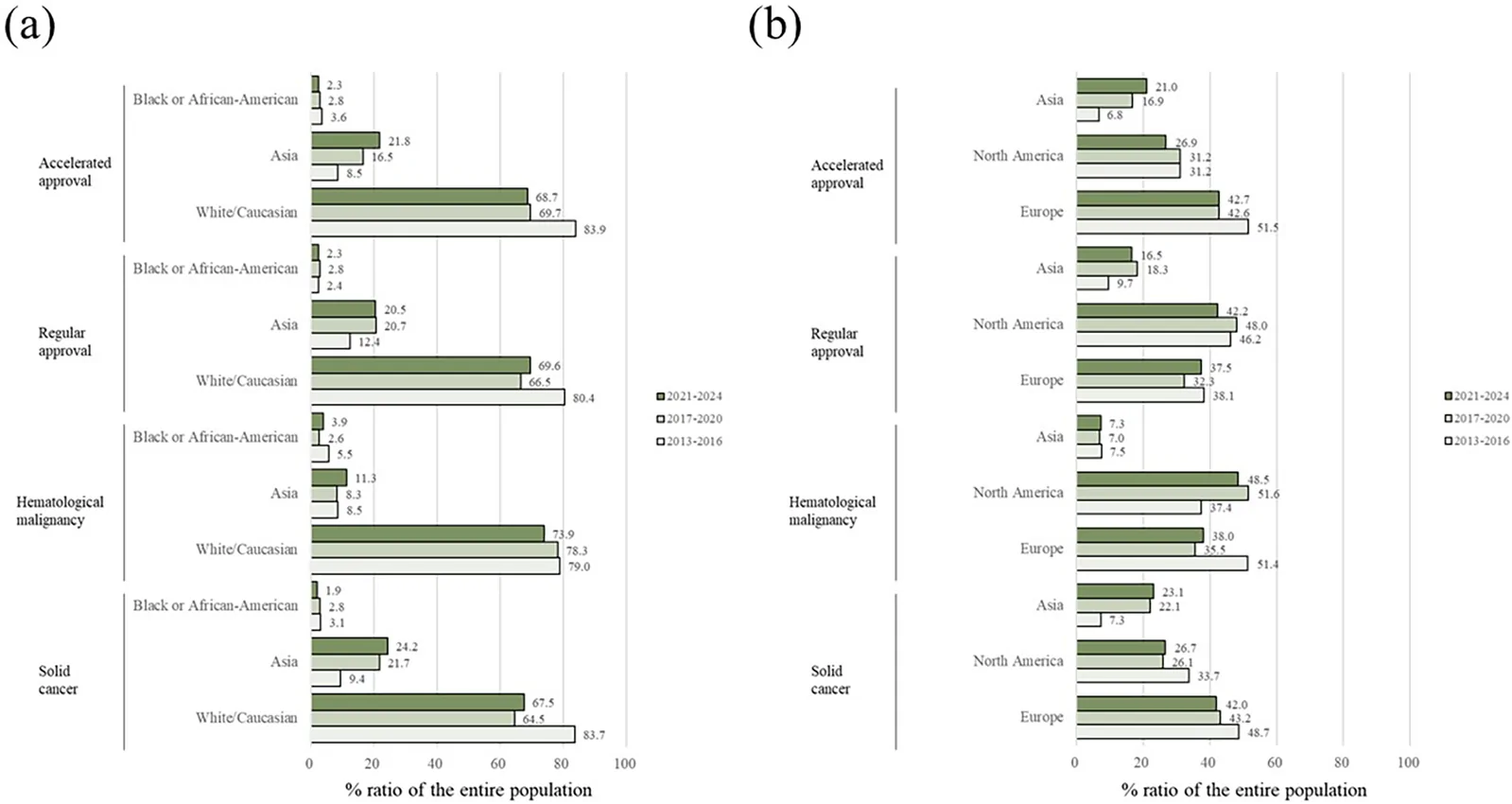
Subgroup analysis of race **(a)** and trial region **(b)** changes.

In the subgroup analysis, the majority race was White for solid cancers, hematological malignancies, regular approvals, and accelerated approvals (Figure 3). The proportion of Asians and the Asian region increased in trials related to solid cancers and regular approvals. Black people had a slightly higher representation in trials related to hematological malignancies in 2013–2017 (5.5%), while in other subgroups, their proportion was below 4%.

**Figure 4:**
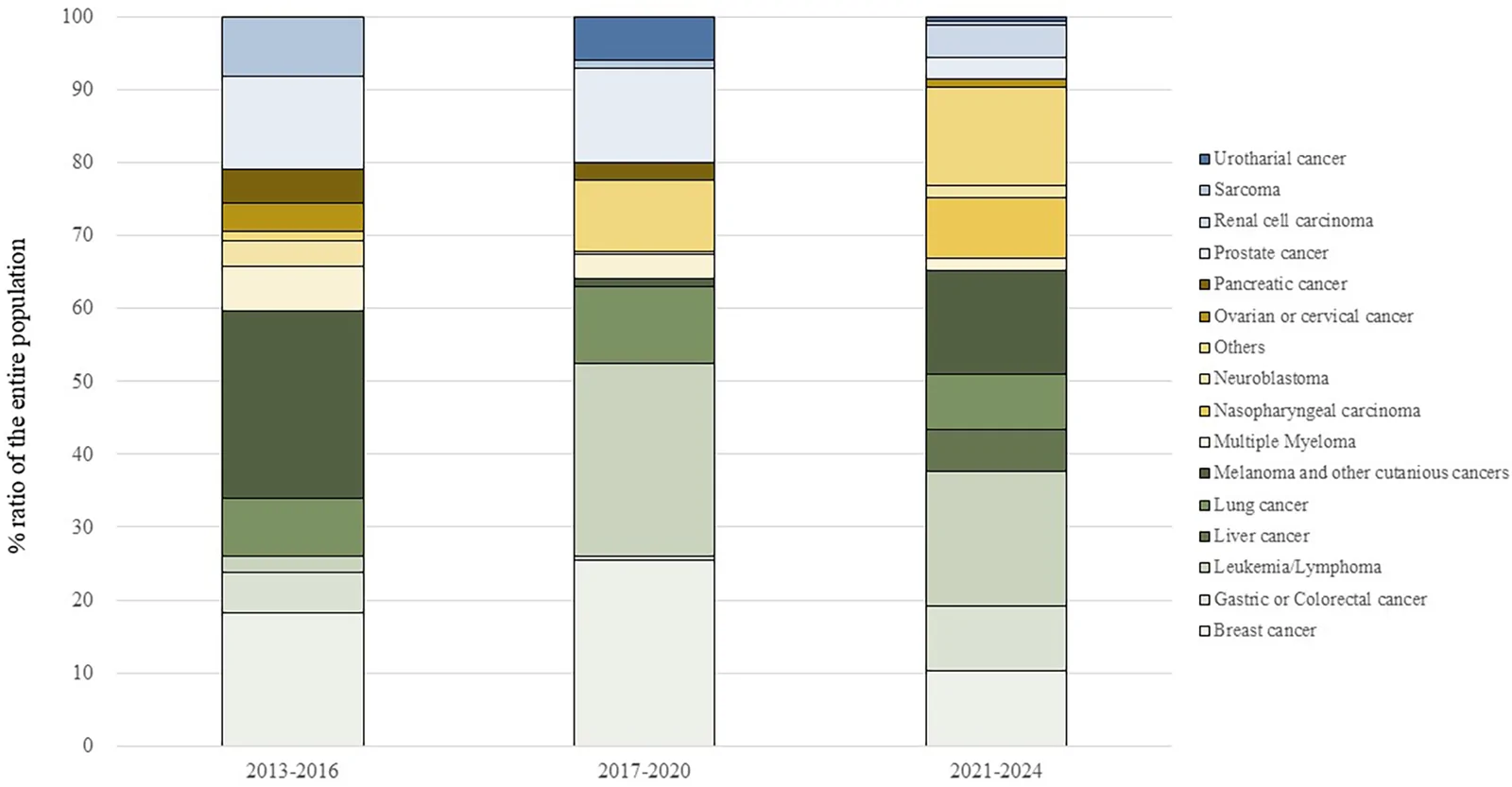
Ratio of each cancer type during the study periods.

To understand the impact on individual diseases, we addressed the contribution of each cancer type in 2013–2016, 2017–2020, and 2021–2024. However, we observed no contribution from any specific cancer type (Figure 4).

## 4 Discussion

In this study, we report on the trial population change in FDA-approved cancer drugs by race and trial region in 2013–2024. Our results indicated that the majority race remained White from 83.2% in 2013–2016 through 68.9% in 2021–2024. This result may be partly explained by the geographic distribution of trial enrolment, as North America accounted for 34.1% in 2013–2016 and 31.4% in 2021–2024 and Europe accounted for 48.9% in 2013–2016 and 41.1% in 2021–2024. Asians increased from 9.3% to 21.4% from 2013 to 2016 through 2021–2024. In parallel, participants from Asia increased from 7.4% to 19.7% during the period. Black people accounted for 3.3% or less of the total trial participants during the period.

The interpretation of these findings in race depends on the benchmark used to assess trial representation. Although many trials supporting FDA oncology approvals are conducted globally, these datasets are submitted to support regulatory decisions for medical products intended for use in the United States. Therefore, US population-based benchmarks provide a relevant regulatory and public health context for interpreting the representation of historically underrepresented groups in FDA oncology approval datasets, particularly Black people. A previous race analysis of clinical trials leading to FDA oncology drug approvals between 2008 and 2018 compared the racial composition of trial participants with US population-based cancer incidence and mortality estimates and reported that Black people were underrepresented (6). Moreover, similar trend was observed in specific cancer field. Another study reported non-Hispanic Black people are underrepresented compared to non-Hispanic White participants when enrollment is benchmarked to the U.S. female population with gynecological cancer (12). Consistent with these prior reports, our results indicated that Black people (3.3% or less) remained underrepresented compared with US population-based benchmarks [the United States Census reported that Black people account for 13.5% of the US population at population estimates Jul 1 2025 (13)]. The persistently low enrollment of Black people is particularly important in the context of ongoing FDA and scientific efforts to improve diversity in clinical trials (2–5, 7). This suggests that factors other than FDA guidance and expert recommendations may be associated with persistent racial underrepresentation in oncology clinical trials. Relevant barriers may include clinician, patients, trials and institutions barriers (14). As the latest Diversity Action Plan of the FDA became effective in June 2024, its impact on actual enrollment in clinical trials should be studied in the future (7).

In contrast, the increase in Asian participants and Asia-region trial enrollment should be interpreted separately from Black people underrepresentation. The increase in the proportions of Asian participants and participants enrolled in Asia may reflect changes in global oncology drug development and the increasing contribution of Asia-region trials to FDA oncology approval datasets. This increase may be attributed to several factors in the clinical trial environment in Asia. First, the number of clinical trials recruiting in Asia has increased (15). This expansion may have increased opportunities for Asian patients to participate in oncology trials. Second, continued urbanization may improve access to oncology clinical trials, because trial participation is often influenced by proximity to large hospitals or cancer centers, and rural patients may face greater geographic and transportation barriers to trial participation (16). Third, clinical trial infrastructure in Asia has expanded. For example, China — the largest country in Asia — has more than 200 cancer-specialized hospitals (17). Traditionally, there were limited awareness and trust in cancer screening and clinical trials in China; however, a clinical trial registry has now been initiated (17–19). For Japan, the standpoint of the Pharmaceuticals and Medical Devices Agency changed in 2023. The agency now recommends that a phase 1 study is not required in Japan unless deemed necessary before enrolling Japanese participants in global studies (20). This recommendation may reduce delays and start-up burdens for including Japan in global clinical development programs. Overall, the increasing proportions of Asian participants and participants enrolled in Asia suggest the growing role of Asia in global oncology drug development. They indicate that trial populations supporting FDA oncology approvals are changing alongside global shifts in clinical trial activity, infrastructure, and regulatory strategy. Sustainable drug development strategy in the 2020s and going forward may warrant further research considering the perspective of retaining both diversity and feasibility in clinical trials.

The present study had some limitations. First, a major limitation of this study is the extent of missing race and trial-region data. The included data packages accounted for only 54% of the eligible approval notifications, because race or trial-region composition was not publicly reported in the FDA review documents or approval notifications for the remaining approvals. This missingness may affect the generalizability of our findings, particularly if trials without publicly available race or trial-region data differed systematically from included trials in terms of sponsor, study region, cancer type, trial design, or reporting practices. Therefore, the present findings should be interpreted as trends among FDA oncology approval datasets with publicly available race and trial-region information, rather than as a complete description of all trials supporting FDA oncology approvals (Figure 1). Additionally, the recruitment of participants by race based on disease incidence could provide researchers with a deeper understanding; however, this study did not address cancer incidence by race (4). Finally, our analysis was limited to clinical trial data packages supporting FDA-approved cancer drugs in the United States from 2013 to 2024. Therefore, trials conducted globally that were not submitted for FDA approval may show different enrollment trends.

In conclusion, this study quantified changes in race and trial region demographics in cancer trials supporting FDA approvals from 2013 to 2024. The results showed that Black people remain underrepresented relative to their proportion in the US population, whereas the enrollment of Asian participants and participants enrolled from Asia countries has increased. These findings should be interpreted in the context of the changes in the Asian population and the clinical and regulatory circumstances in Asian countries.

## Data Availability

The original contributions presented in the study are included in the article/Supplementary Material, further inquiries can be directed to the corresponding author.
