# Supplementary material for: Race and trial region changes in cancer clinical trials between 2013 and 2024
Source: Front Med (Lausanne). 2026 Sep 9;13:1894533. doi: 10.3389/fmed.2026.1894533 (PMC13598081; doi:10.3389/fmed.2026.1894533)
Supplement: Supplementary file 1 [file Table1.docx]

| Table S1. Oncology (cancer)/hematologic malignancies approval notifications \| FDA | | | | | | | |
| --- | --- | --- | --- | --- | --- | --- | --- |
| Description | **Date** | **Type of approval** | **Study name** | **Original indication** | **Middle category** | **High category** |  |
| FDA approved ado-trastuzumab emtansine (KADCYLA for injection, Genentech, Inc.) for use as a single agent for the treatment of patients with HER2-positive, metastatic breast cancer who previously received trastuzumab and a taxane, separately or in combination. Patients should have either received prior therapy for metastatic disease or developed disease recurrence during or within 6 months of completing adjuvant therapy. | 2/22/2013 | RA | BO21976 | Breast cancer | Breast cancer | Solid |  |
| FDA approved radium Ra 223 dichloride (Xofigo Injection, Bayer HealthCare Pharmaceuticals Inc.) for the treatment of patients with castration-resistant prostate cancer, symptomatic bone metastases, and no known visceral metastatic disease. Xofigo is an alpha particle emitting a radiotherapeutic drug that mimics calcium and forms complexes with hydroxyapatite at areas of increased bone turnover, such as bone metastases. | 5/15/2013 | RA | BCI-06 | Prostate cancer | Prostate cancer | Solid |  |
| FDA approved trametinib (MEKINIST tablet, GlaxoSmithKline, LLC) for the treatment of patients with unresectable or metastatic melanoma with BRAF V600E or V600K mutations as detected by an FDA-approved test. | 5/29/2013 | RA | MEK114267 | Melanoma | Melanoma and other cutaneous cancers | Solid |  |
| FDA approved dabrafenib (TAFINLAR capsule, GlaxoSmithKline, LLC) for the treatment of patients with unresectable or metastatic melanoma with BRAF V600E mutation as detected by an FDA-approved test. | 5/29/2013 | RA | BRF113683 | Melanoma | Melanoma and other cutaneous cancers | Solid |  |
| FDA approved ramucirumab (Cyramza, Eli Lilly and Company) for use as a single agent for the treatment of patients with advanced or metastatic, gastric or gastroesophageal junction (GEJ) adenocarcinoma with disease progression on or after prior treatment with fluoropyrimidine- or platinum-containing chemotherapy. Ramucirumab is a recombinant monoclonal antibody of the IgG1 class that binds to vascular endothelial growth factor receptor-2 (VEGFR-2), blocking its activation. | 4/21/2014 | RA | REGARD | Gastric cancer | Gastric or Colorectal cancer | Solid |  |
| FDA approved siltuximab (Sylvant Injection Janssen Biotech, Inc.) for the treatment of patients with multicentric Castleman’s disease (MCD) who are human immunodeficiency virus (HIV-) -negative and human herpes virus-8 (HHV-8) -negative. | 4/23/2014 | RA | CNTO328MCD2001 | Multicentric Castleman’s disease | Others | Hematologic malignancy |  |
| FDA granted accelerated approval to ceritinib (ZYKADIA, Novartis Pharmaceuticals Corporation) for the treatment of patients with anaplastic lymphoma kinase (ALK)-positive, metastatic non-small cell lung cancer (NSCLC) with disease progression on or who are intolerant to crizotinib. | 4/29/2014 | AA | X2101 | Lung cancer | Lung cancer | Solid |  |
| FDA granted accelerated approval to pembrolizumab (KEYTRUDA, Merck Sharp & Dohme Corp.) for the treatment of patients with unresectable or metastatic melanoma and disease progression following ipilimumab and, if BRAF V600 mutation-positive, a BRAF inhibitor. | 9/4/2014 | AA | P001 | Melanoma | Melanoma and other cutaneous cancers | Solid |  |
| FDA-approved olaparib capsules (Lynparza, AstraZeneca Pharmaceuticals LP) as monotherapy for the treatment of patients with deleterious or suspected deleterious germline BRCA mutated (gBRCAm) (as detected by an FDA-approved test) advanced ovarian cancer who have been treated with three or more prior lines of chemotherapy. | 12/19/2014 | RA | Study 42 | Ovarian cancer | Ovarian or cervical cancer | Solid |  |
| FDA granted accelerated approval to nivolumab (OPDIVO, Bristol-Myers Squibb Company) for the treatment of patients with unresectable or metastatic melanoma and disease progression following ipilimumab and, if BRAF V600 mutation-positive, a BRAF inhibitor. | 12/22/2014 | AA | CA209037 | Melanoma | Melanoma and other cutaneous cancers | Solid |  |
| FDA granted accelerated approval to palbociclib (IBRANCE, Pfizer, Inc.) for use in combination with letrozole for the treatment of postmenopausal women with estrogen receptor (ER)-positive, human epidermal growth factor receptor 2 (HER2)-negative advanced breast cancer as initial endocrine-based therapy for metastatic disease. | 2/3/2015 | AA | PALOMA-1 | Breast cancer | Breast cancer | Solid |  |
| FDA granted approval to nivolumab (OPDIVO, Bristol-Myers Squibb Company) for the treatment of patients with metastatic squamous non-small cell lung cancer (NSCLC) with progression on or after platinum-based chemotherapy. | 3/4/2015 | RA | CA209063 | Lung cancer | Lung cancer | Solid |  |
| FDA approved dinutuximab (Unituxin, United Therapeutics Corporation), in combination with granulocyte-macrophage colony-stimulating factor (GM-CSF), interleukin-2 (IL-2), and 13-cis-retinoic acid (RA), for the treatment of pediatric patients with high-risk neuroblastoma who achieve at least a partial response to prior first-line multiagent, multimodality therapy. | 3/10/2015 | RA | Study 301 | Neuroblastoma | Neuroblastoma | Solid |  |
| FDA approved sonidegib (Odomzo Capsules, Novartis Pharmaceuticals Corporation) for the treatment of patients with locally advanced basal cell carcinoma (BCC) that has recurred following surgery or radiation therapy, or those who are not candidates for surgery or radiation therapy. | 7/24/2015 | RA | A2201 | Basal cell carcinoma | Melanoma and other cutaneous cancers | Solid |  |
| FDA approved irinotecan liposome injection (ONIVYDE, Merrimack Pharmaceuticals, Inc.), administered in combination with fluorouracil (5FU) and leucovorin (LV), for the treatment of patients with metastatic adenocarcinoma of the pancreas whose disease has progressed following gemcitabine-based therapy. | 10/22/2015 | RA | NAPOLI-1 | Adenocarcinoma | Pancreatic cancer | Solid |  |
| FDA approved trabectedin (Yondelis Injection, Janssen) for the treatment of patients with unresectable or metastatic liposarcoma or leiomyosarcoma who have received a prior anthracycline-containing regimen. | 10/23/2015 | RA | Study 3007 | Liposarcoma or leiomyosarcoma | Sarcoma | Solid |  |
| FDA approved cobimetinib (COTELLIC Tablets, Genentech, Inc.) for the treatment of patients with unresectable or metastatic melanoma with BRAF V600E or V600K mutation, in combination with vemurafenib. Cobimetinib is not indicated for the treatment of patients with wild-type BRAF melanoma. | 11/10/2015 | RA | GO28141 | Melanoma | Melanoma and other cutaneous cancers | Solid |  |
| FDA granted accelerated approval to daratumumab injection (DARZALEX, Janssen Biotech, Inc.), administered as a single agent for the treatment of patients with multiple myeloma who have received at least three prior lines of therapy, including a proteasome inhibitor (PI) and an immunomodulatory agent, or who are double-refractory to a PI and an immunomodulatory agent. | 11/16/2015 | AA | Integrated | Multiple Myeloma | Multiple Myeloma | Hematologic malignancy |  |
| FDA approved elotuzumab (EMPLICITI, Bristol-Myers Squibb Company) in combination with lenalidomide and dexamethasone for the treatment of patients with multiple myeloma who have received one to three prior therapies. | 11/30/2015 | RA | CA204009 | Multiple Myeloma | Multiple Myeloma | Hematologic malignancy |  |
| FDA granted accelerated approval to alectinib (ALECENSA capsules, Hoffmann-La Roche Inc.) for the treatment of patients with anaplastic lymphoma kinase (ALK)-positive metastatic non-small cell lung cancer (NSCLC) who have progressed on or are intolerant to crizotinib. | 12/11/2015 | AA | NP28673 | Lung cancer | Lung cancer | Solid |  |
| FDA approved venetoclax (VENCLEXTA tablets, marketed by AbbVie, Inc. and Genentech USA, Inc.) for the treatment of patients with chronic lymphocytic leukemia (CLL) with 17p deletion, as detected by an FDA-approved test, who have received at least one prior therapy. | 4/11/2016 | RA | M13-982 | Leukemia | Leukemia/lymphoma | Hematologic malignancy |  |
| FDA granted accelerated approval to rucaparib (RUBRACA, Clovis Oncology, Inc.) for the treatment of patients with deleterious BRCA mutation (germline and/or somatic) associated with advanced ovarian cancer who have been treated with two or more chemotherapies. | 12/19/2016 | AA | ARIEL2 | Ovarian cancer | Ovarian or cervical cancer | Solid |  |
| FDA granted accelerated approval to avelumab (BAVENCIO, EMD Serono, Inc.) for patients with locally advanced or metastatic urothelial carcinoma whose disease progressed during or following platinum-containing chemotherapy or within 12 months of neoadjuvant or adjuvant platinum-containing chemotherapy. | 5/9/2017 | AA | Study 001 | Urothelial carcinoma | Urothelial cancer | Solid |  |
| FDA granted regular approval to the combination of rituximab and hyaluronidase human (RITUXAN HYCELA, Genentech Inc.) for adult patients with follicular lymphoma, diffuse large B-cell lymphoma, and chronic lymphocytic leukemia. | 6/22/2017 | RA | MabEase | No specific cancer | Others | Solid |  |
| FDA granted regular approval to enasidenib (IDHIFA, Celgene Corp.) for the treatment of adult patients with relapsed or refractory acute myeloid leukemia with an isocitrate dehydrogenase-2 (IDH2) mutation as detected by an FDA-approved test. | 8/1/2017 | RA | Integrated | Leukemia | Leukemia/lymphoma | Hematologic malignancy |  |
| FDA granted regular approval to a liposome-encapsulated combination of daunorubicin and cytarabine (VYXEOS, Jazz Pharmaceuticals, Inc.) for the treatment of adults with newly diagnosed therapy-related AML (t-AML) or AML with myelodysplasia-related changes (AML-MRC) – two types of AML having a poor prognosis. | 8/3/2017 | RA | Study 301 | AML | Leukemia/lymphoma | Hematologic malignancy |  |
| FDA approved inotuzumab ozogamicin (BESPONSA, Wyeth Pharmaceuticals Inc., a subsidiary of Pfizer Inc.) for the treatment of adults with relapsed or refractory B-cell precursor acute lymphoblastic leukemia (ALL). | 8/17/2017 | RA | Study 1022 | Leukemia | Leukemia/lymphoma | Hematologic malignancy |  |
| FDA approved abemaciclib (VERZENIO, Eli Lilly and Company) in combination with fulvestrant for women with HR-positive, HER2-negative advanced or metastatic breast cancer with disease progression following endocrine therapy. | 9/28/2017 | RA | MONARCH 2 | Breast cancer | Breast cancer | Solid |  |
| FDA granted regular approval to axicabtagene ciloleucel (YESCARTA, Kite Pharma, Inc.) for the treatment of adult patients with relapsed or refractory large B-cell lymphoma after two or more lines of systemic therapy, including diffuse large B-cell lymphoma (DLBCL) not otherwise specified, primary mediastinal large B-cell lymphoma, high-grade B-cell lymphoma, and DLBCL arising from follicular lymphoma. | 10/18/2017 | RA | ZUMA-1 | Lymphoma | Leukemia/Lymphoma | Hematologic malignancy |  |
| FDA approved lutetium Lu 177 dotatate (LUTATHERA, Advanced Accelerator Applications USA, Inc.) — a radiolabeled somatostatin analog — for the treatment of somatostatin receptor-positive gastroenteropancreatic neuroendocrine tumors (GEP-NETs), including foregut, midgut, and hindgut neuroendocrine tumors in adults. | 1/26/2018 | RA | NETTER-1 | Pancreatic cancer | Pancreatic cancer | Solid |  |
| FDA approves apalutamide for non-metastatic castration-resistant prostate cancer. | 2/14/2018 | RA | SPARTAN | Prostate cancer | Prostate cancer | Solid |  |
| FDA-approved abemaciclib (VERZENIO, Eli Lilly and Company) in combination with an aromatase inhibitor as initial endocrine-based therapy for postmenopausal women with hormone receptor (HR)-positive, human epidermal growth factor receptor 2 (HER2)-negative advanced or metastatic breast cancer. | 2/26/2018 | RA | MONARCH 3 | Breast cancer | Breast cancer | Solid |  |
| FDA-approved ivosidenib (Tibsovo, Agios Pharmaceuticals, Inc.) for adult patients with relapsed or refractory acute myeloid leukemia (AML) with a susceptible IDH1 mutation as detected by an FDA-approved test. | 7/20/2018 | RA | AG120-C-001 | Leukemia | Leukemia/lymphoma | Hematologic malignancy |  |
| FDA-approved dacomitinib tablets (VIZIMPRO, Pfizer Pharmaceutical Company) for the first-line treatment of patients with metastatic non-small cell lung cancer (NSCLC) with epidermal growth factor receptor (EGFR) exon 19 deletion or exon 21 L858R substitution mutations as detected by an FDA-approved test. | 9/27/2018 | RA | ARCHER 1050 | Lung cancer | Lung cancer | Solid |  |
| FDA-approved cemiplimab-rwlc (LIBTAYO, Regeneron Pharmaceuticals Inc.) for patients with metastatic cutaneous squamous cell carcinoma (CSCC) or locally advanced CSCC who are not candidates for curative surgery or curative radiation. | 9/28/2018 | RA | Integrated | Squamous cell carcinoma | Melanoma and other cutaneous cancers | Solid |  |
| FDA granted accelerated approval to lorlatinib (LORBRENA, Pfizer, Inc.) for patients with anaplastic lymphoma kinase (ALK)-positive metastatic non-small cell lung cancer (NSCLC) whose disease has progressed on crizotinib and at least one other ALK inhibitor for metastatic disease or whose disease has progressed on alectinib or ceritinib as the first ALK inhibitor therapy for metastatic disease. | 11/2/2018 | AA | B7461001 | Lung cancer | Lung cancer | Solid |  |
| FDA-approved glasdegib (DAURISMO, Pfizer Labs) in combination with low-dose cytarabine (LDAC), for newly-diagnosed acute myeloid leukemia (AML) in patients who are 75 years old or older or who have comorbidities that preclude intensive induction chemotherapy. | 11/21/2018 | RA | B1371003 | AML | Leukemia/Lymphoma | Hematologic malignancy |  |
| FDA-approved calaspargase pegol-mknl (ASPARLAS, Servier Pharmaceuticals LLC) — an asparagine-specific enzyme — as a component of a multi-agent chemotherapeutic regimen for acute lymphoblastic leukemia (ALL) in pediatric and young adult patients aged 1 month to 21 years. This new product allows a longer interval between doses compared to other available pegaspargase products. | 12/20/2018 | RA | DFCI 11-001 | Leukemia | Leukemia/lymphoma | Hematologic malignancy |  |
| FDA-approved trastuzumab and hyaluronidase-oysk injection for subcutaneous use (Herceptin Hylecta, Genentech Inc.). Herceptin Hylecta is a combination of trastuzumab — a HER2/neu receptor antagonist — and hyaluronidase — an endoglycosidase — for the treatment of HER2-overexpressing breast cancer. | 2/28/2019 | RA | HannaH | Breast cancer | Breast cancer | Solid |  |
| FDA granted accelerated approval to erdafitinib (BALVERSA, Janssen Pharmaceutical Companies) for patients with locally advanced or metastatic urothelial carcinoma, with susceptible FGFR3 or FGFR2 genetic alterations, that has progressed during or following platinum-containing chemotherapy, including within 12 months of neoadjuvant or adjuvant platinum-containing chemotherapy. | 4/12/2019 | AA | BLC2001 | Urothelial carcinoma | Urothelial cancer | Solid |  |
| FDA-approved alpelisib (PIQRAY, Novartis Pharmaceuticals Corporation) in combination with fulvestrant for postmenopausal women, and men, with hormone receptor (HR)-positive, human epidermal growth factor receptor 2 (HER2)-negative, PIK3CA-mutated, advanced or metastatic breast cancer as detected by an FDA-approved test following progression on or after an endocrine-based regimen. | 5/24/2019 | RA | SOLAR-1 | Breast cancer | Breast cancer | Solid |  |
| FDA granted accelerated approval to polatuzumab vedotin-piiq (POLIVY, Genentech, Inc.) — a CD79b-directed antibody-drug conjugate — indicated in combination with bendamustine and a rituximab product for adult patients with relapsed or refractory diffuse large B-cell lymphoma (DLBCL), not otherwise specified, after at least two prior therapies. | 6/10/2019 | AA | GO29365 | Lymphoma | Leukemia/lymphoma | Hematologic malignancy |  |
| FDA granted accelerated approval to entrectinib (ROZLYTREK, Genentech Inc.) for adults and pediatric patients aged 12 years and older with solid tumors that have a neurotrophic tyrosine receptor kinase (NTRK) gene fusion without a known acquired resistance mutation, who are metastatic or for whom surgical resection is likely to result in severe morbidity, and who have progressed following treatment or have no satisfactory standard therapy. | 8/15/2019 | AA | Integrated | Lung cancer | Lung cancer | Solid |  |
| FDA approved fedratinib (INREBIC, Impact Biomedicines, Inc.) for adults with intermediate-2 or high-risk primary or secondary (post-polycythemia vera or post-essential thrombocythemia) myelofibrosis (MF). | 8/16/2019 | RA | JAKARTA | Myelofibrosis | Others | Hematologic malignancy |  |
| FDA granted accelerated approval to zanubrutinib (BRUKINSA, BeiGene, Ltd.) for adult patients with mantle cell lymphoma (MCL) who have received at least one prior therapy. | 11/14/2019 | AA | BGB-3111-206 | Lymphoma | Leukemia/Lymphoma | Hematologic malignancy |  |
| FDA granted accelerated approval to enfortumab vedotin-ejfv (PADCEV, Astellas Pharma US, Inc.) for adult patients with locally advanced or metastatic urothelial cancer who have previously received a programmed death receptor-1 (PD-1) or programmed death-ligand 1 (PD-L1) inhibitor, and a platinum-containing chemotherapy in the neoadjuvant/adjuvant, locally advanced or metastatic setting. | 12/18/2019 | AA | EV-201 | Urothelial carcinoma | Urothelial cancer | Solid |  |
| FDA granted accelerated approval to fam-trastuzumab deruxtecan-nxki (ENHERTU, Daiichi Sankyo) for patients with unresectable or metastatic HER2-positive breast cancer who have received two or more prior anti-HER2-based regimens in the metastatic setting. | 12/20/2019 | AA | DS8201-A-U201 | Breast cancer | Breast cancer | Solid |  |
| FDA approved avapritinib (AYVAKIT, Blueprint Medicines Corporation) for adults with unresectable or metastatic gastrointestinal stromal tumor (GIST) harboring a platelet-derived growth factor receptor alpha (PDGFRA) exon 18 mutation, including D842V mutations. | 1/9/2020 | RA | BLU-285-1101. | Gastrointestinal stromal tumor | Gastric or colorectal cancer | Solid |  |
| FDA granted accelerated approval to tazemetostat (TAZVERIK, Epizyme, Inc.) for adults and pediatric patients aged 16 years and older with metastatic or locally advanced epithelioid sarcoma not eligible for complete resection. | 1/23/2020 | AA | EZH-202 | Epithelioid sarcoma | Sarcoma | Solid |  |
| FDA approved isatuximab-irfc (SARCLISA, Sanofi-aventis U.S. LLC) in combination with pomalidomide and dexamethasone for adult patients with multiple myeloma who have received at least two prior therapies, including lenalidomide and a proteasome inhibitor. | 3/2/2020 | RA | EFC14335 | Multiple myeloma | Multiple myeloma | Hematologic malignancy |  |
| FDA approved selumetinib (KOSELUGO, AstraZeneca) for pediatric patients, aged 2 years and older, with neurofibromatosis type 1 (NF1) who have symptomatic, inoperable plexiform neurofibromas (PN). | 4/10/2020 | RA | SPRINT | Neurofibromatosis type 1 | Others | Solid |  |
| FDA approved mitomycin (JELMYTO™, UroGen Pharma) for adult patients with low-grade upper tract urothelial cancer (LG-UTUC). | 4/15/2020 | RA | UT-001 | Urothelial cancer | Urothelial cancer | Solid |  |
| FDA granted accelerated approval to sacituzumab govitecan-hziy (TRODELVY, Immunomedics, Inc.) for adult patients with metastatic triple-negative breast cancer who received at least two prior therapies for metastatic disease. | 4/22/2020 | AA | IMMU-132-01 | Breast cancer | Breast cancer | Solid |  |
| FDA granted accelerated approval to lurbinectedin (ZEPZELCA, Pharma Mar S.A.) for adult patients with metastatic small cell lung cancer (SCLC) with disease progression on or after platinum-based chemotherapy. June 15, 2020 | 6/15/2020 | AA | PM1183-B-005-14 | Lung cancer | Lung cancer | Solid |  |
| FDA approved an oral combination of decitabine and cedazuridine (INQOVI, Astex Pharmaceuticals, Inc.) for adult patients with myelodysplastic syndromes (MDS), including the following: | 7/7/2020 | RA | Integrated | Myelodysplastic syndromes | Leukemia/lymphoma | Hematologic malignancy |  |
| FDA granted accelerated approval to brexucabtagene autoleucel (TECARTUS, Kite, a Gilead Company) — a CD19-directed genetically modified autologous T cell immunotherapy — for the treatment of adult patients with relapsed or refractory mantle cell lymphoma (MCL). | 7/24/2020 | AA | ZUMA-2 | Lymphoma | Leukemia/lymphoma | Hematologic malignancy |  |
| FDA granted accelerated approval to tafasitamab-cxix (MONJUVI, MorphoSys US Inc.) — a CD19-directed cytolytic antibody — indicated in combination with lenalidomide for adult patients with relapsed or refractory diffuse large B-cell lymphoma (DLBCL) not otherwise specified — including DLBCL arising from low-grade lymphoma — and who are ineligible for autologous stem cell transplant. | 7/31/2020 | AA | L-MIND | Lymphoma | Leukemia/lymphoma | Hematologic malignancy |  |
| FDA approved azacitidine tablets (ONUREG, Celgene Corporation) for continued treatment of patients with acute myeloid leukemia who achieved first complete remission (CR) or complete remission with incomplete blood count recovery (CRi) following intensive induction chemotherapy and are unable to complete intensive curative therapy. | 9/1/2020 | RA | CC-486-AML-001 | AML | Leukemia/lymphoma | Hematologic malignancy |  |
| FDA granted accelerated approval to naxitamab (DANYELZA, Y-mAbs Therapeutics, Inc.) in combination with granulocyte-macrophage colony-stimulating factor (GM-CSF) for pediatric patients aged 1 year and older and adult patients with relapsed or refractory high-risk neuroblastoma in the bone or bone marrow demonstrating a partial response, minor response, or stable disease compared to prior therapy. | 11/25/2020 | AA | 12-230 | Neuroblastoma | Neuroblastoma | Solid |  |
| FDA granted accelerated approval to tepotinib (Tepmetko, EMD Serono Inc.) for adult patients with metastatic non-small cell lung cancer (NSCLC) harboring mesenchymal-epithelial transition (MET) exon 14 skipping alterations. | 2/3/2021 | AA | VISION | Lung cancer | Lung cancer | Solid |  |
| FDA approved lisocabtagene maraleucel (Breyanzi, Juno Therapeutics, Inc.) for the treatment of adult patients with relapsed or refractory (R/R) large B-cell lymphoma after two or more lines of systemic therapy, including diffuse large B-cell lymphoma (DLBCL) not otherwise specified (including DLBCL arising from indolent lymphoma), high-grade B-cell lymphoma, primary mediastinal large B-cell lymphoma, and follicular lymphoma grade 3B. February 5, 2021 | 2/5/2021 | RA | Study 17001 | Lymphoma | Leukemia/lymphoma | Hematologic malignancy |  |
| FDA approved tivozanib (Fotivda, AVEO Pharmaceuticals, Inc.) — a kinase inhibitor — for adult patients with relapsed or refractory advanced renal cell carcinoma (RCC) following two or more prior systemic therapies. March 10, 2021 | 3/10/2021 | RA | TIVO-3 | Renal cell carcinoma | Renal cell carcinoma | Solid |  |
| FDA approved idecabtagene vicleucel (Abecma, Bristol Myers Squibb) for the treatment of adult patients with relapsed or refractory multiple myeloma after four or more prior lines of therapy, including an immunomodulatory agent, a proteasome inhibitor, and an anti-CD38 monoclonal antibody. This is the first FDA-approved cell-based gene therapy for multiple myeloma. March 26, 2021 | 3/26/2021 | RA | MM-001 | Multiple myeloma | Multiple myeloma | Hematologic malignancy |  |
| On April 23, 2021, the FDA granted accelerated approval to loncastuximab tesirine-lpyl (Zynlonta, ADC Therapeutics SA) — a CD19-directed antibody and alkylating agent conjugate — for adult patients with relapsed or refractory large B-cell lymphoma after two or more lines of systemic therapy, including diffuse large B-cell lymphoma (DLBCL) not otherwise specified, DLBCL arising from low-grade lymphoma, and high-grade B-cell lymphoma. | 4/23/2021 | AA | ADCT‐402‐201 | Lymphoma | Leukemia/lymphoma | Hematologic malignancy |  |
| FDA granted accelerated approval to infigratinib (Truseltiq, QED Therapeutics, Inc.) — a kinase inhibitor — for adults with previously treated, unresectable locally advanced or metastatic cholangiocarcinoma with a fibroblast growth factor receptor 2 (FGFR2) fusion or other rearrangement as detected by an FDA-approved test. | 5/28/2021 | AA | CBGJ398X2204 | Cholangiocarcinoma | Liver cancer | Solid |  |
| FDA granted accelerated approval to sotorasib (Lumakras™, Amgen, Inc.) — a RAS GTPase family inhibitor — for adult patients with KRAS G12C mutated locally advanced or metastatic non-small cell lung cancer (NSCLC), as determined by an FDA-approved test, who have received at least one prior systemic therapy. | 5/28/2021 | AA | Study 20170543 | Lung cancer | Lung cancer | Solid |  |
| FDA approved asparaginase erwinia chrysanthemi (recombinant)-rywn) (Rylaze, Jazz Pharmaceuticals, Inc.) as a component of a multi-agent chemotherapeutic regimen for the treatment of acute lymphoblastic leukemia (ALL) and lymphoblastic lymphoma (LBL) in adult and pediatric patients aged 1 month or older who have developed hypersensitivity to E. coli-derived asparaginase. | 7/1/2021 | RA | JZP458- 201 | Leukemia | Leukemia/lymphoma | Hematologic malignancy |  |
| FDA granted accelerated approval to mobocertinib (Exkivity, Takeda Pharmaceuticals, Inc.) for adult patients with locally advanced or metastatic non-small cell lung cancer (NSCLC) with epidermal growth factor receptor (EGFR) exon 20 insertion mutations, as detected by an FDA-approved test, whose disease has progressed on or after platinum-based chemotherapy. | 9/15/2021 | AA | Integrated | Lung cancer | Lung cancer | Solid |  |
| On September 20, 2021, the FDA granted accelerated approval to tisotumab vedotin-tftv (Tivdak, Seagen Inc.) — a tissue factor-directed antibody and microtubule inhibitor conjugate — for adult patients with recurrent or metastatic cervical cancer with disease progression on or after chemotherapy. | 9/20/2021 | AA | GCT1015-04 | Cervical cancer | Ovarian or cervical cancer | Solid |  |
| On November 22, 2021, the FDA approved sirolimus protein-bound particles for injectable suspension (albumin-bound) (Fyarro, Aadi Bioscience, Inc.) for adult patients with locally advanced unresectable or metastatic malignant perivascular epithelioid cell tumor (PEComa). | 11/23/2021 | RA | Integrated | Perivascular epithelioid cell tumor | Others | Solid |  |
| On January 25, 2022, the FDA approved tebentafusp-tebn (Kimmtrak, Immunocore Limited) — a bispecific gp100 peptide-HLA-directed CD3 T cell engager — for HLA-A*02:01-positive adult patients with unresectable or metastatic uveal melanoma. | 1/25/2022 | RA | Study 202 | Uveal melanoma | Melanoma and other cutaneous cancers | Solid |  |
| On March 18, 2022, the FDA approved nivolumab and relatlimab-rmbw (Opdualag, Bristol-Myers Squibb Company) for adult and pediatric patients aged 12 years or older with unresectable or metastatic melanoma. Opdualag is a fixed-dose combination of the LAG-3-blocking antibody relatlimab and the programmed death receptor-1 blocking antibody nivolumab. | 3/18/2022 | RA | CA224047 | Melanoma | Melanoma and other cutaneous cancers | Solid |  |
| On September 30, 2022, the FDA granted accelerated approval to futibatinib (Lytgobi, Taiho Oncology, Inc.) for adult patients with previously treated, unresectable, locally advanced, or metastatic intrahepatic cholangiocarcinoma harboring fibroblast growth factor receptor 2 (FGFR2) gene fusions or other rearrangements. | 9/30/2022 | AA | TAS‐120‐101 | Intrahepatic cholangiocarcinoma | Liver cancer | Solid |  |
| On November 10, 2022, the FDA approved tremelimumab (Imjudo, AstraZeneca Pharmaceuticals) in combination with durvalumab (Imfinzi, AstraZeneca Pharmaceuticals) and platinum-based chemotherapy for adult patients with metastatic non-small cell lung cancer (NSCLC) with no sensitizing epidermal growth factor receptor (EGFR) mutation or anaplastic lymphoma kinase (ALK) genomic tumor aberrations. | 11/10/2022 | RA | POSEIDON | Solid tumor | Others | Solid |  |
| On November 14, 2022, the FDA granted accelerated approval to mirvetuximab soravtansine-gynx (Elahere, ImmunoGen, Inc.) for adult patients with folate receptor alpha (FRα) positive, platinum-resistant epithelial ovarian, fallopian tube, or primary peritoneal cancer, who have received one to three prior systemic treatment regimens. Mirvetuximab soravtansine-gynx is a folate receptor alpha-directed antibody and microtubule inhibitor conjugate. Patients are selected for therapy based on an FDA-approved test. | 11/14/2022 | AA | SORAYA | Solid tumor | Others | Solid |  |
| On December 16, 2022, the FDA approved nadofaragene firadenovec-vncg (Adstiladrin, Ferring Pharmaceuticals) for adult patients with high-risk Bacillus Calmette-Guérin (BCG) unresponsive non-muscle invasive bladder cancer (NMIBC) with carcinoma in situ (CIS) with or without papillary tumors. | 12/16/2022 | RA | rAd-IFN-CS-003 | Non-muscle invasive bladder cancer | Urothelial cancer | Solid |  |
| On December 22, 2022, the FDA granted accelerated approval to mosunetuzumab-axgb (Lunsumio, Genentech, Inc.) — a bispecific CD20-directed CD3 T-cell engager — for adult patients with relapsed or refractory follicular lymphoma (FL) after two or more lines of systemic therapy. | 12/23/2022 | AA | GO29781 | Lymphoma | Leukemia/lymphoma | Hematologic malignancy |  |
| On January 27, 2023, the FDA (FDA) approved elacestrant (Orserdu, Stemline Therapeutics, Inc.) for postmenopausal women or adult men with ER-positive, HER2-negative, ESR1-mutated advanced or metastatic breast cancer with disease progression following at least one line of endocrine therapy. | 1/27/2023 | RA | RAD1901-308 | Breast cancer | Breast cancer | Solid |  |
| On March 22, 2023, the FDA granted accelerated approval to retifanlimab-dlwr (Zynyz, Incyte Corporation) for adult patients with metastatic or recurrent locally advanced Merkel cell carcinoma (MCC). | 3/22/2023 | AA | PODIUM-201 | Merkel cell carcinoma | Others | Solid |  |
| On April 17, 2023, the FDA approved omidubicel-onlv (Omisirge, Gamida Cell Ltd.) for use in adult and pediatric patients (aged 12 years and older) with hematologic malignancies who are planned for umbilical cord blood transplantation following myeloablative conditioning to reduce the time to neutrophil recovery and the incidence of infection. | 4/17/2023 | RA | Integrated | Hematologic malignancy | Others | Others |  |
| On May 19, 2023, the FDA granted accelerated approval to epcoritamab-bysp (Epkinly, Genmab US, Inc.) for relapsed or refractory diffuse large B-cell lymphoma (DLBCL) not otherwise specified, including DLBCL arising from indolent lymphoma, and high-grade B-cell lymphoma after two or more lines of systemic therapy. | 5/19/2023 | AA | GCT3013-01 | Lymphoma | Leukemia/lymphoma | Hematologic malignancy |  |
| On June 15, 2023, the FDA granted accelerated approval to glofitamab-gxbm (Columvi, Genentech, Inc.) for relapsed or refractory diffuse large B-cell lymphoma, not otherwise specified (DLBCL, NOS) or large B-cell lymphoma (LBCL) arising from follicular lymphoma, after two or more lines of systemic therapy. | 6/16/2023 | AA | NP30179 | Lymphoma | Leukemia/lymphoma | Hematologic malignancy |  |
| On August 11, 2023, the FDA approved the fixed-dose combination of niraparib and abiraterone acetate (Akeega, Janssen Biotech, Inc.), with prednisone, for adult patients with deleterious or suspected deleterious BRCA-mutated castration-resistant prostate cancer (mCRPC), as determined by an FDA-approved test. | 8/11/2023 | RA | MAGNITUDE | Prostate cancer | Prostate cancer | Solid |  |
| On October 27, 2023, the FDA approved toripalimab-tpzi (LOQTORZ, Coherus BioSciences, Inc.) with cisplatin and gemcitabine for the first-line treatment of adults with metastatic or recurrent, locally advanced nasopharyngeal carcinoma (NPC). FDA also approved toripalimab-tpzi as a single agent for adults with recurrent, unresectable, or metastatic NPC with disease progression on or after platinum-containing chemotherapy. | 10/27/2023 | RA | Integrated | Nasopharyngeal carcinoma | Nasopharyngeal carcinoma | Solid |  |
| On November 8, 2023, the FDA approved fruquintinib (Fruzaqla, Takeda Pharmaceuticals, Inc.) for adult patients with metastatic colorectal cancer (mCRC) who received prior fluoropyrimidine-, oxaliplatin-, and irinotecan-based chemotherapy, an anti-VEGF therapy, and, if RAS wild-type and medically appropriate, an anti-EGFR therapy. | 11/8/2023 | RA | FRESCO-2 | Colorectal cancer | Gastric or colorectal cancer | Solid |  |
| On December 13, 2023, the FDA approved eflornithine (IWILFIN, USWM, LLC) to reduce the risk of relapse in adult and pediatric patients with high-risk neuroblastoma (HRNB) who have demonstrated at least a partial response to prior multi-agent, multimodality therapy, including anti-GD2 immunotherapy. | 12/13/2023 | RA | Strata | Neuroblastoma | Neuroblastoma | Solid |  |
| On February 16, 2024, the FDA granted accelerated approval to lifileucel (Amtagvi, Iovance Biotherapeutics, Inc.) — a tumor-derived autologous T cell immunotherapy — for adult patients with unresectable or metastatic melanoma previously treated with a PD-1 blocking antibody, and if BRAF V600 positive, a BRAF inhibitor with or without a MEK inhibitor. | 2/16/2024 | AA | C-144-01 | Melanoma | Melanoma and other cutaneous cancers | Solid |  |
| On June 6, 2024, the FDA approved imetelstat (Rytelo, Geron Corporation) — an oligonucleotide telomerase inhibitor — for adults with low- to intermediate-1 risk myelodysplastic syndromes (MDS) with transfusion-dependent anemia requiring four or more red blood cell units over 8 weeks who have not responded to or have lost response to or are ineligible for erythropoiesis-stimulating agents (ESAs). | 6/6/2024 | RA | MDS-3001 | Myelodysplastic syndromes | Leukemia/lymphoma | Hematologic malignancy |  |
| On August 2, 2024, the FDA granted accelerated approval to afamitresgene autoleucel (TECELRA, Adaptimmune, LLC) — a melanoma-associated antigen A4 (MAGE-A4)-directed genetically modified autologous T cell immunotherapy — for adults with unresectable or metastatic synovial sarcoma who have received prior chemotherapy, are HLA-A*02:01P, -A*02:02P, -A*02:03P, or -A*02:06P positive, and whose tumor expresses the MAGE-A4 antigen as determined by FDA-approved or cleared companion diagnostic devices. | 8/2/2024 | AA | ADP-0044-002 | Synovial sarcoma | Sarcoma | Solid |  |
| On October 10, 2024, the FDA approved inavolisib (Itovebi, Genentech, Inc.) with palbociclib and fulvestrant for adults with endocrine-resistant, PIK3CA-mutated, hormone receptor (HR)-positive, human epidermal growth-factor receptor 2 (HER2)-negative, locally advanced or metastatic breast cancer, as detected by an FDA-approved test, following recurrence on or after completing adjuvant endocrine therapy. | 10/10/2024 | RA | WO41554 | Breast cancer | Breast cancer | Solid |  |
| On November 8, 2024, the FDA approved obecabtagene autoleucel (Aucatzyl, Autolus Inc.) — a CD19-directed genetically modified autologous T cell immunotherapy — for adults with relapsed or refractory B-cell precursor acute lymphoblastic leukemia (ALL). | 11/8/2024 | RA | FELIX | Leukemia | Leukemia/lymphoma | Hematologic malignancy |  |
| On November 20, 2024, the FDA granted accelerated approval to zanidatamab-hrii (Ziihera, Jazz Pharmaceuticals, Inc.) — a bispecific HER2-directed antibody — for previously treated, unresectable or metastatic HER2-positive (IHC 3+) biliary tract cancer (BTC), as detected by an FDA-approved test. | 11/21/2024 | AA | Integrated | Biliary tract cancer | Liver cancer | Solid |  |

RA; Regular approval, AA; Accelerated approval, Integrated represents integrated population from some studies including PIVOTAL.
